# Sensorimotor control of gait: a novel approach for the study of the interplay of visual and proprioceptive feedback

**DOI:** 10.3389/fnhum.2015.00014

**Published:** 2015-02-09

**Authors:** Ryan Frost, Jeffrey Skidmore, Marco Santello, Panagiotis Artemiadis

**Affiliations:** ^1^Human-Oriented Robotics and Control Lab, School for Engineering of Matter Transport and Energy, Arizona State UniversityTempe, AZ, USA; ^2^Neural Control of Movement Laboratory, School of Biological and Healthy Systems Engineering, Arizona State UniversityTempe, AZ, USA

**Keywords:** gait, visual feedback, proprioceptive feedback, gait rehabilitation, rehabilitation robotics

## Abstract

Sensorimotor control theories propose that the central nervous system exploits expected sensory consequences generated by motor commands for movement planning, as well as online sensory feedback for comparison with expected sensory feedback for monitoring and correcting, if needed, ongoing motor output. In our study, we tested this theoretical framework by quantifying the functional role of expected vs. actual proprioceptive feedback for planning and regulation of gait in humans. We addressed this question by using a novel methodological approach to deliver fast perturbations of the walking surface stiffness, in conjunction with a virtual reality system that provided visual feedback of upcoming changes of surface stiffness. In the “predictable” experimental condition, we asked subjects to learn associating visual feedback of changes in floor stiffness (sand patch) during locomotion to quantify kinematic and kinetic changes in gait prior to and during the gait cycle. In the “unpredictable” experimental condition, we perturbed floor stiffness at unpredictable instances during the gait to characterize the gait-phase dependent strategies in recovering the locomotor cycle. For the “unpredictable” conditions, visual feedback of changes in floor stiffness was absent or inconsistent with tactile and proprioceptive feedback. The investigation of these perturbation-induced effects on contralateral leg kinematics revealed that visual feedback of upcoming changes in floor stiffness allows for both early (preparatory) and late (post-perturbation) changes in leg kinematics. However, when proprioceptive feedback is not available, the early responses in leg kinematics do not occur while the late responses are preserved although in a, slightly attenuated form. The methods proposed in this study and the preliminary results of the kinematic response of the contralateral leg open new directions for the investigation of the relative role of visual, tactile, and proprioceptive feedback on gait control, with potential implications for designing novel robot-assisted gait rehabilitation approaches.

## Introduction

The etymology of the word “Anthropos”, the Greek word for Human, includes one of the defining characteristics of human beings, which is the ability to stand upright and walk. Locomotion is one of the most important sensorimotor behaviors in humans that has enabled important evolutionary behaviors covering a wide range of interactions with the environment, including survival and exploration. From the perspective of neural control and biomechanics, the control of gait requires kinematic and dynamic coordination of the limbs and muscles, multi-sensory fusion, and robust control mechanisms.

Locomotion results from intricate dynamic interactions between a central “program” and feedback mechanisms. The central program relies fundamentally on a genetically determined spinal circuit capable of generating basic locomotion patterns, as well as neural drive through various descending pathways that can trigger, stop, and/or steer locomotion. Sensory feedback from muscle and skin afferents, as well as other sensory modalities (vision, audition, vestibular), dynamically adapt the locomotion pattern to the requirements of the environment (Rossignol et al., 2006). Recent work has stressed the importance of the interaction between peripheral sensory information (Field-Fote and Dietz, 2007) and descending inputs from the motor cortex (Yang and Gorassini, 2006) in shaping the Central Pattern Generator (Grillner, 2003) function, and particularly in guiding post-lesional plasticity mechanisms. It should be noted that a spinal pattern generator does not appear to be sufficient to control over-ground walking. Supraspinal control is needed to provide both the drive for locomotion as well as sensorimotor integration needed to negotiate a complex environment (Norton, 2010). Available evidence highlights the importance of supraspinal pathways for the control of bipedal walking (Nielsen, 2003; Forrester et al., 2008) and the way sensory feedback shapes motor learning in the brain (Petersen et al., 1998).

Previous investigations of the role of afferent sensory feedback in gait control mechanisms has used sensory perturbations. Various platforms and protocols have been used to investigate reflex mechanisms during different phases of the gait, with the majority of the experimental protocols focusing on over-ground walking and dropping of the supportive surfaces at distinct gait phases (Nakazawa et al., 2004; van der Linden et al., 2007). Although many studies have focused on the effect of unilateral perturbations delivered to ipsilateral leg muscles, several studies have also investigated bilateral responses (Dietz et al., 1989; Nakazawa et al., 2004; van der Linden et al., 2007). During posture maintenance, powerful unilateral displacement of one leg have been shown to elicit bilateral responses both in adults and healthy human infants (Berger et al., 1984, 1987; Lam et al., 2003). Moreover, the disruption of load feedback as well as the length of specific muscles during walking under perturbations have been associated with evoked muscular activations of the leg (Dietz and Duysens, 2000; Sinkjaer et al., 2000; Boyer and Nigg, 2007; af Klint et al., 2010; Klarner, 2010).

While the importance of visual feedback in human gait is well established (Patla, 1998; Rossignol et al., 2006), how vision affects gait and its interactions with other gait mechanisms is not well understood (Egerton et al., 2011). Whereas most studies have focused on the effects of visual perturbations on gait during either altered visual flow (Mohler et al., 2007) or obstacle avoidance (Rhea and Rietdyk, 2007), a few studies have investigated the interplay of multiple sensory modalities. Perry et al. (2001) systematically manipulated vision and cutaneous sensation to investigate the contributions of these two sensory modalities and found that both play a phase-specific role in gait termination. These authors further suggested that visual information is involved in slowing down the forward progression of the center of mass of the walker and in guiding final foot placement, while plantar-surface mechanoreceptors provide sensory feedback about the foot contact to initiate braking forces. Prokop et al. (1997) found that a combination of visual and proprioceptive information is necessary for modifying walking velocity and suggest that visual information modifies stride length while proprioceptive feedback maintains a stable stride frequency, leading to a change in walking velocity.

Despite the significant insights provided by these studies, there is a gap in our understanding of the interplay of visual and proprioceptive control mechanisms in human gait, specifically in our understanding of anticipatory control mechanisms driven by visual feedback. To address this gap, we investigated the effects of expected vs. actual proprioceptive feedback through the use of fast perturbations of the walking surface stiffness in combination with visual feedback provided through a virtual reality system. In the “predictable” experimental condition, subjects experienced walking surface compliance (both rigid and low stiffness) that was consistent with visual feedback. In the “unpredictable” experimental condition, the floor stiffness was perturbed at unpredictable instances during gait while visual feedback of changes in floor compliance was absent or inconsistent with tactile and proprioceptive feedback. This experimental approach allowed us to quantify the relative roles of visual and proprioceptive feedback on gait control.

## Methods

### Experimental setup

#### Variable stiffness treadmill (VST)

**Figure 1:**
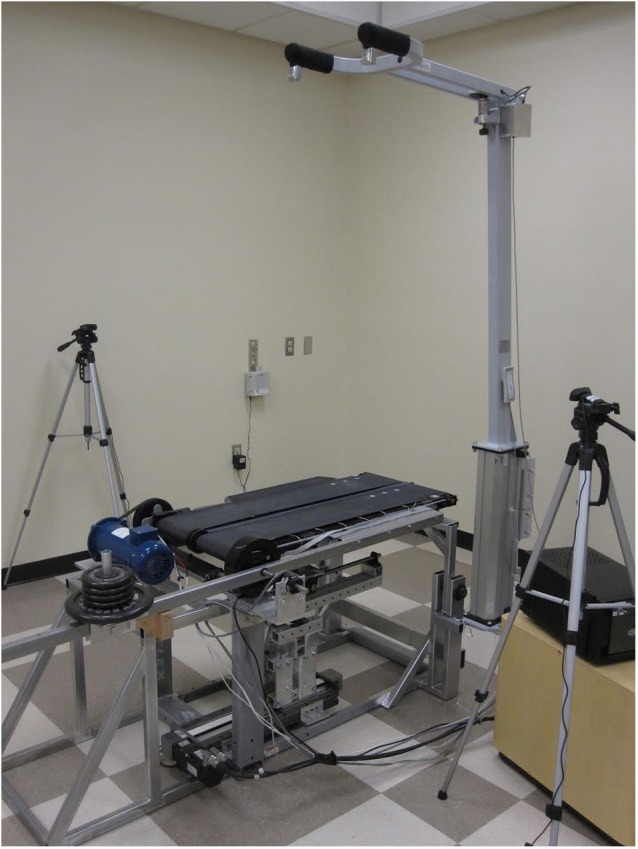
**Variable Stiffness Treadmill (VST)**.

**Figure 2:**
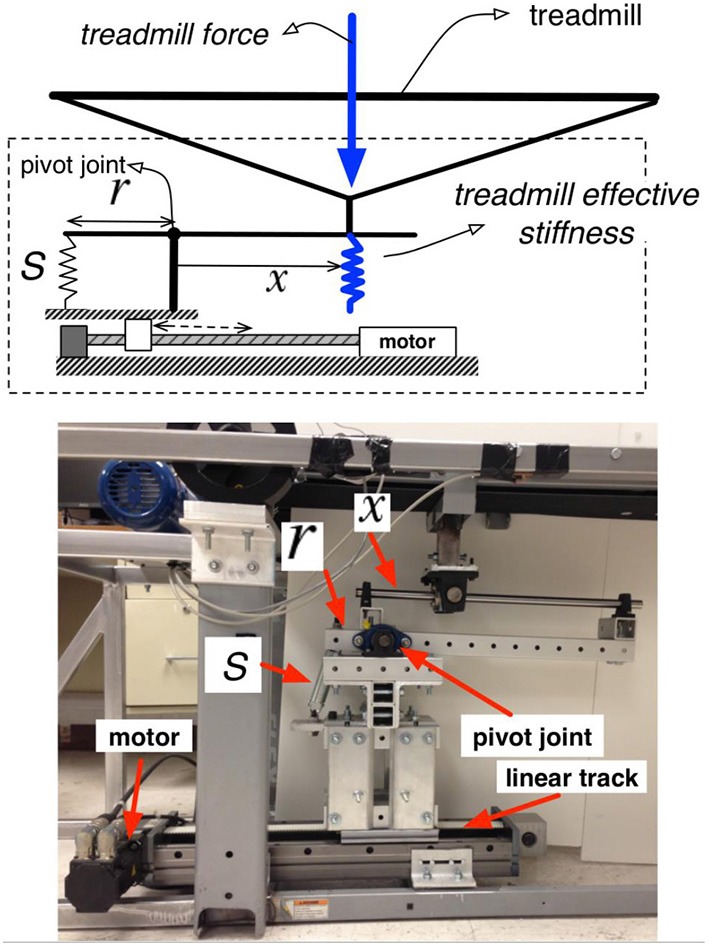
**Variable stiffness mechanism**.

The basis of the experimental protocol was the variable stiffness treadmill (VST; Skidmore et al., 2014a). The VST (Figure 1) is a split-track treadmill capable of altering the floor stiffness of the left track independently of the right. This is accomplished by a variable stiffness mechanism that is located underneath the treadmill belt. This mechanism can be controlled in real-time using external feedback (e.g., the walker’s foot position). The mechanism can vary the stiffness of the treadmill in the range of 61.7 N/m to near-infinite (>1 MN/m) stiffness in 130 ms, with the accuracy of 30 N/m. The device’s ability to change of the stiffness near-instantaneously and very accurately prevents the subject from anticipating stiffness changes based on a preceding vibration/noise from the variable stiffness mechanism. Figure 2 depicts the variable stiffness mechanism. Variable floor stiffness is achieved by moving the linear track, which results in changing the moment arm (*x*), the amount of force required to extend the spring (*S*), and thus controls the effective floor stiffness. The distance (r) between the spring and the pivot point does not change. More details can be found at Skidmore et al. (2014a).

#### Body weight support (BWS)

While walking on the treadmill, subjects were provided with bodyweight support (BWS) using a custom-made device built by LiteGait. The system is capable of supporting the entire weight of the subjects, or a portion of the subject’s weight. The subject wears a harness similar to those used for rock-climbing, which buckles in to straps hanging down from an overhead beam. Two load sensors separate the straps from the beam, allowing both the weight supported and the left-right distribution of the weight supported to be calculated. In addition to providing support against gravity, the harness helped ensure that the subject remained centered on the treadmill. This measure was necessary due to the subject’s inability to visually sense the location of the treadmill when walking in the virtual world (see below).

#### Leg motion tracking

**Figure 3:**
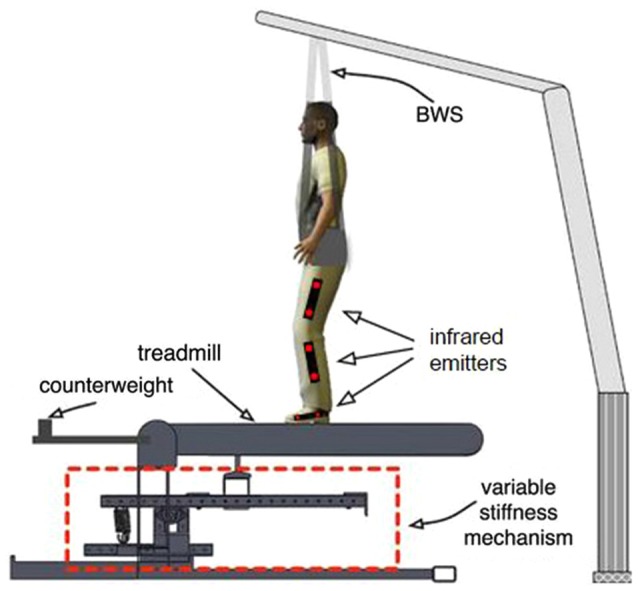
**Experimental setup**. The area outlined in red is the variable stiffness mechanism outlined in Figure 2. The figure shows the location of infrared emitters that are detected by two tripod-mounted cameras shown in Figure 1.

Two high-speed (200 frames per second) infrared cameras (Code Laboratories Inc, model: DUO MINI LX) were used to track the position of infrared emitters (Super Bright LEDs Inc, model: IR-1WS-850) attached to the subject’s legs. The two cameras were placed on the left and right side of the subject, facing inward to the subject’s legs. Six markers were placed on each leg facing laterally outward. Two markers were placed in line with the bones on each thigh, shank and foot, in order to be aligned with the three main segments of the legs. The positions of the markers were used to calculate the hip, knee, and ankle joint angles for both legs in real time. The virtual reality system sampled the calculated joint angles every 60 ms (16.7 Hz) for display in the visual interface. The positions of the two markers on the left foot were averaged to determine the position of the subject’s left foot along the treadmill (Figure 3) which was used in the feedforward controller and to determine when heel strike (HS) and toe-off (TO) occur.

#### Virtual reality headset

The visual stimuli were presented to the subject using a virtual reality headset (Oculus Virtual Reality, model: Rift Development Kit 1). The headset contains a 1280 × 800 LCD, and one lens per eye. The screen is split in half, so that each eye sees a slightly shifted image. This provides the user with a stereoscopic view of the media sent to the device, offering three-dimensional depth perception. The headset also tracks the rotational orientation of the user’s head and alters the virtual view accordingly. The stereoscopic sense of depth and the view tracking gave subjects the feeling of immersion in a custom designed virtual environment.

#### Virtual environment

**Figure 4:**
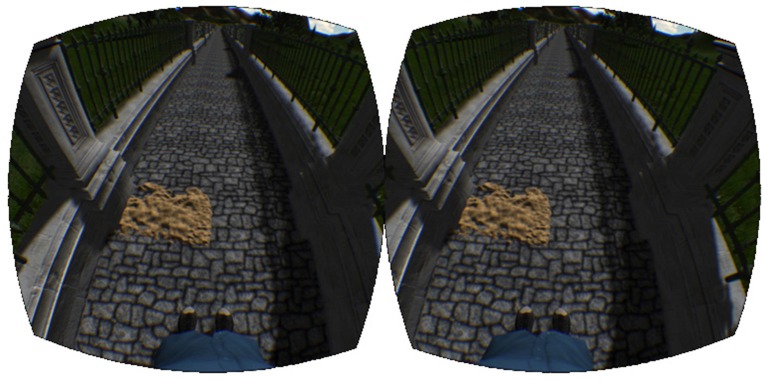
**Virtual reality environment**. The virtual reality environment consisting of the walkway, sand patch, and virtual avatar.

The virtual walking environment was designed with the game development package Unity3D^1^ using free game-objects provided with the software. The virtual world consisted of a cobblestone pathway, fenced in on both sides (Figure 4). Mountains, trees, and tall grass were added for immersive effect. The environment was scaled to realistic sizes, so that walking one meter on the treadmill looked and felt like walking one meter in the virtual world. The speed of the treadmill was automatically synchronized with the movement of the camera view in the environment. In addition, the recorded joint angles were used to control the legs of a virtual avatar. Subjects were given a first-person view of the avatar and, looking down, were able to watch their virtual legs move in time with their actual legs (Figure 4).

### Experimental protocol

Along the virtual walkway (Figure 4), patches of sand were placed at random intervals on the left side of the path. The locations of these patches were generated randomly each time the experiment was run based on the following three criteria: 20 m of walking before the first sand patch, 6 ± 1 m between each patch, and a total of 120 patches altogether.

**Figure 5:**
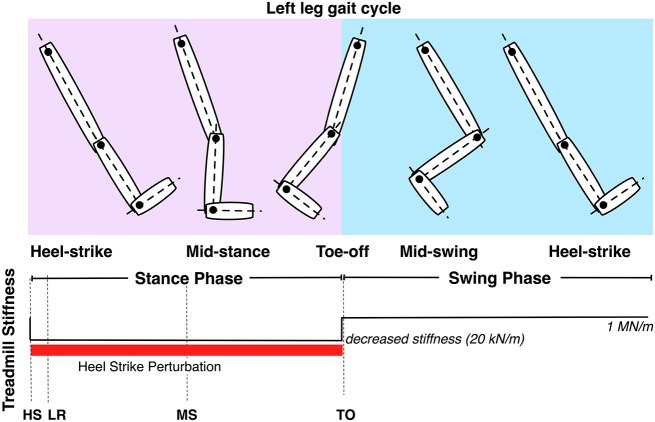
**Controllable treadmill stiffness throughout the left leg gait cycle**. The stiffness perturbation start at heel-strike (HS) of the left leg and ends at the toe-off (TO) of the left leg. LR and MS stand for loading response and mid-stance of the left leg.

The VST was used to effect changes in floor stiffness to the left track of the treadmill, in conjunction with visual stimuli. The perturbations consisted of a change from 1 MN/m to 20 kN/m, starting at left HS, enduring through the left stance phase and ending at left toe off (Figure 5).

In order to match the subject’s visual expectations with the change in stiffness, two conditions had to be met for a stiffness change to occur. First, the virtual avatar’s left foot had to break the vertical plane of the nearest edge of the sand patch. The second condition required that the subject’s actual, physical left foot was moving forward. Once both of these conditions were met, the VST would lower the floor stiffness upon the next HS of the left foot, and return to infinite stiffness upon toe off. This design allowed subjects to see that when the virtual avatar stepped on a sand patch, the floor stiffness would be decreased.

### Perturbation types

We delivered three types of perturbations: visual and physical (VP), visual only (VO), or physical only (PO). Out of the 120 patches we used, we delivered 100 VP perturbations, 10 VO perturbations, and 10 PO perturbations.

VP perturbations were used as control perturbations to train subjects to associate stepping on sand patches with a drop in floor stiffness. VO perturbations were used to quantify the kinematics of the perturbed and unperturbed leg in response to an expected reduction in floor stiffness. The visual appearance of the sand patch was identical for VP and VO perturbations, the only difference between these two conditions being that for the VO perturbation, floor stiffness was not changed when the subject stepped on the sand patch. The PO perturbation was used to quantify the kinematic response of the perturbed and unperturbed leg to a reduction of floor stiffness change but without visual warning. This was accomplished by inducing a stiffness change halfway between two patches. Visually, the sand patch would appear normal, but the treadmill controller was triggered as if the sand patch was halfway between its actual location and the previous patch. Hence, in the PO perturbation, a stiffness change would happen without a patch being located underneath the subject’s foot.

Subject performed one trial consisting of a total of 120 perturbations as described above. As most perturbations were VP (83%), subjects could learn to associate visually-cued sand patches with a drop in stiffness. However, all perturbations (VP, VO, and PO) were presented in random order using the following design. For the 10th patch, subjects experience either a VO or PO perturbation, followed by a PO or VO perturbation every 6 ± 2 patches. Within the trial, each subject experienced 10 VO and 10 PO perturbations.

Four healthy subjects (ages: 22 ± 3 yrs; 3 males, 1 female) walked on the treadmill at a speed of 0.70 m/s while wearing the virtual reality headset. Subjects walked between 620 and 860 m over the course of the experiment, depending on the random distances between consecutive patches. Subjects were provided with 30% bodyweight support. Before starting to walk, subjects were given approximately 30–60 s to look around the virtual world and familiarize with the virtual avatar’s legs and congruence with their own movements. Once the subject informed the experimenter that he/she was ready, the treadmill was brought up to speed and the virtual avatar began moving towards the first sand patch. After walking over all 120 sand patches, the treadmill was slowed to a stop, and the subject was allowed to dismount the treadmill. All subjects gave informed consent for the experimental protocol approved by the Arizona State University Institutional Review Board.

## Results

Although the left leg was directly perturbed through the left treadmill stiffness change, our analyses focused on the effects of the perturbation on the contralateral (right) leg response. Our previous work has shown that the ipsilateral-perturbed leg kinematics are significantly affected by the stiffness perturbation (Artemiadis and Krebs, 2011a,b; Skidmore et al., 2014b), thus a comparison between the perturbed and unperturbed gait cycle is redundant and beyond the scope of the present study. Moreover, the responses related to visual feedback usually occur late (~200 ms after the perturbation), and are therefore not easily detectable in the perturbed leg gait cycle.

**Figure 6:**
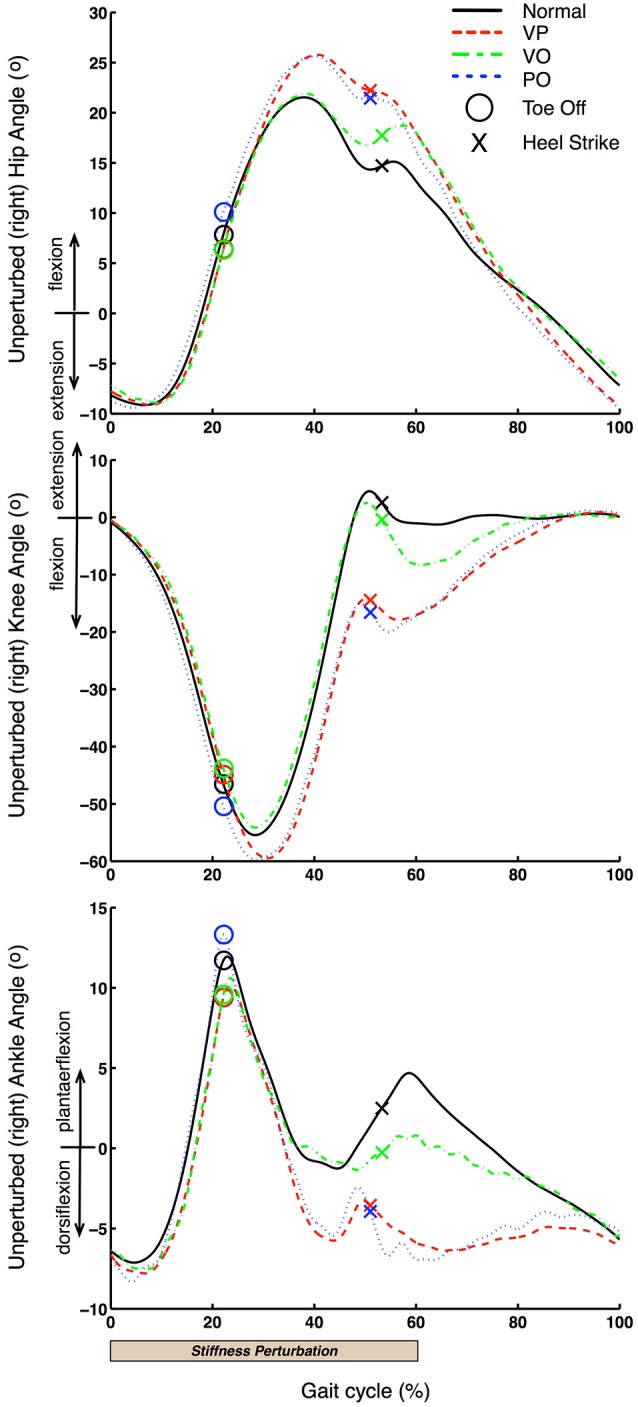
**Unperturbed leg kinematics**. Unperturbed (right) leg kinematics is shown for one representative subject. 0% gait cycle corresponds to the beginning of the left leg perturbation, which is close to the terminal stance phase of the right leg.

First, we analyzed the hip flexion-extension, knee flexion-extension and ankle dorsi-plantar flexion angles of the right leg. The gait cycles were categorized in four categories, according to the four conditions identified: Normal (unperturbed), VP, VO and PO. As shown in Figure 5, the stiffness perturbations started at the HS of the left leg, and ended at the TO of the same leg. When the left leg was at HS, the right leg was at terminal stance phase. The gait cycle of the right leg was defined as the starting point at the onset of the perturbation (HS of the left leg) to quantify the effects of the perturbation more precisely. The kinematics of the right leg in the four different conditions are shown in Figure 6 showing joint angles averaged over approximately 20 cycles per condition from a representative subject. The TO and HS events of the right leg across the four different conditions were identified based on the kinematic data (Figure 6). The duration of the left leg stiffness perturbation was approximately 60% of the average gait cycle (HS to TO), corresponding to a duration of ~1.4 s. The duration of the perturbation was consistent across all perturbation conditions in order to generate consistent data that would always include a full stance phase.

**Figure 7:**
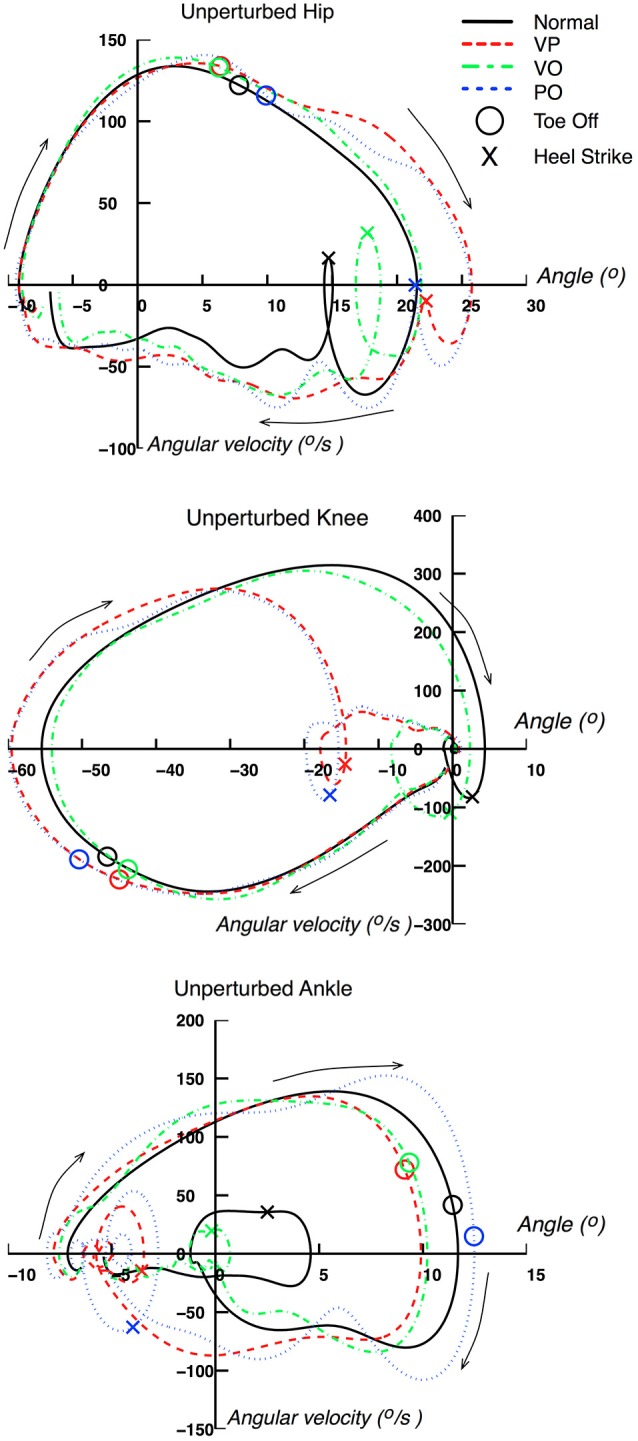
**Phase space of unperturbed leg kinematics**. Unperturbed (right) leg kinematics represented in phase space (i.e., angular position vs. angular velocity). Data are from a representative subject.

The kinematics of the right leg were also represented in phase space (angular position vs. angular velocity). This space offers a better understanding of the behavior of a periodic system after a perturbation, and allows the analysis of the stability and robustness of the system in general. Angular velocities for all three joints investigated were computed by differentiating the angular position data, after low-pass filtering them (Butterworth filter, 2nd order, cut-off frequency of 4 Hz). The phase plots of each of the three joints of the right leg are shown in Figure 7. The TO and HS for each of the four conditions are shown. The data shown and the definition of the right cycle gait cycle is identical to the one used for Figure 6.

## Conclusions—discussion

Figure 6 shows that VP and PO conditions have very similar effects on the right (unperturbed) leg. Specifically, both hip and knee joint kinematics are significantly affected right after the right leg starts the swing phase at ~280 ms (20% of the average gait cycle of 1.4 s) after the start of the perturbation delivered to the left leg. This latency is consistent with our previous studies (Artemiadis and Krebs, 2011a,b; Skidmore et al., 2014b). Most importantly, these observations support the hypothesis that inter-leg coordination involves supraspinal pathways, which would account for the long delay in the response of the non-perturbed response (~280 ms). Additionally, a similar response but a longer latency (~420 ms) relative to the onset of the perturbation is observed at the ankle joint. Thus, it appears that the right leg kinematics respond to the perturbation to the contralateral leg by accelerating the swing phase and bring the foot in contact with the treadmill earlier relative to the unperturbed case. This interpretation is consistent with the earlier HS in VP and PO conditions that is facilitated by an additional flexion of the hip and knee joints, and larger dorsiflexion combined with faster plantar-flexion of the ankle joint (40–50% of the gait cycle; Figure 6).

Figure 6 also shows that there is a kinematic effect of the perturbation on the right leg kinematics also in the VO condition. Therefore, even if there is a visual “warning” of the stiffness perturbation but the perturbation never happens, the contralateral (right) leg kinematics changes in an anticipatory rather than reactive fashion. Specifically, we observe an effect on the kinematics of all joints (hip, knee, ankle) that starts at ~630 ms after the perturbation onset and just before the HS of the right leg. These kinematic effects are similar to the effects observed in the VP and PO conditions, i.e., acceleration of the swing phase and shortened stride length, which can be explained by increased knee flexion and decreased ankle plantarflexion.

Figure 7 provides an additional representation of the effects of the perturbations on the right leg kinematics. The acceleration of the gait cycle evoked by the perturbations for VP and PO conditions can be seen clearly in the phase space representation by following the clockwise rotation denoted by the arrows. The phase space representation of the three leg joints for both VP and PO conditions exhibit a distinct acceleration of joint rotations through a pattern that resembles the normal gait cycle, but shifted earlier in time through the gait cycle. For example, the loop in the knee phase space representation, in the center of the plot where the HS is included, happens earlier in the VP and PO cases, when the knee is still at −20 degrees. A similar behavior can be seen in the hip and ankle joints. It is also worth noting that the phase representation of the VP and PO cases converges to the normal one before the end of the gait cycle, which can also be seen in Figure 6.

For the VO condition, the phase representation provides further insight about two main features: (1) the evoked responses have similar characteristics to the ones associated with VP and PO perturbations, but delayed with respect to the latter; and (2) the kinematics for the VO condition converge to the normal ones within the gait cycle. It should be emphasized that the VO response in phase space resembles that observed for the VP and PO conditions in terms of acceleration profile of the gait cycle, but lies between the normal cycle and the VP and PO cycles in the phase space. The latter is obvious when examining the loop of the hip phase that includes the HS on the right bottom corner of the graph. A similar behavior can be observed in the corresponding loops of the knee and ankle joints.

The presented method of analyzing the interplay between visual and proprioceptive and tactile feedback in gait resulted in important observations. First, when there is no physical perturbation, and therefore proprioceptive feedback is not elicited, visual feedback can evoke contralateral leg responses that resemble those caused by proprioceptive feedback in response to a mechanical perturbation of the opposite leg. This leads to the validation of the hypothesis that a learnt mapping between visual and proprioceptive feedback creates or activates mechanisms, that are probably supraspinally mediated, that control inter-leg coordination.

However, evoked responses associated with only visual feedback of floor stiffness changes (VO) were significantly delayed relative to those caused by a physical perturbation. These data can be interpreted as follows. Visual cues (warning) act to mediate anticipatory/predictive control of gait, however they only evoke *late* responses. These responses appear to be *independent* from proprioceptive feedback, as suggested by time shift of visually-cued responses relative to proprioceptive-dependent responses. Moreover, our results support the existence of only late responses associated with visual feedback of upcoming changes in floor stiffness. This is supported by the observation that in the early phases of the gait cycle, VP and PO responses are almost identical, which suggests that the predictive role of visual feedback does not activate any *early* motor mechanisms.

The results of the present study should be considered as preliminary due to the small sample of subjects. Furthermore, more work is needed to identify the neural mechanisms underlying the observed kinematic responses of the unperturbed leg to mechanical perturbations delivered to the contralateral leg. Nevertheless, our findings are promising as they shed new light on inter-leg coordination mechanisms and open new avenues for research, However, the scope of this paper is to introduce a novel method of investigating the inter-play of visual and proprioceptive feedback in gait. The proposed method, facilitated by a novel and unique technological architecture (the VST setup), can be potentially beneficial not only for understanding sensorimotor control of gait, but also for significantly improving neural rehabilitation protocols for impaired walkers by applying the identified principles and developing model-based protocols for gait therapy.

## Conflict of interest statement

The authors declare that the research was conducted in the absence of any commercial or financial relationships that could be construed as a potential conflict of interest.

## Footnotes

^1^www.unity3d.com
